# Prevention of Dermal Abscess Formation Caused by *Staphylococcus aureus* Using Phage JD007 in Nude Mice

**DOI:** 10.3389/fmicb.2018.01553

**Published:** 2018-07-23

**Authors:** Bingyu Ding, Qingtian Li, Mingquan Guo, Ke Dong, Yan Zhang, Xiaokui Guo, Qingzhong Liu, Li Li, Zelin Cui

**Affiliations:** ^1^Department of Laboratory Medicine, Shanghai General Hospital, Shanghai Jiao Tong University School of Medicine, Shanghai, China; ^2^Department of Laboratory Medicine, Ruijin Hospital, Shanghai Jiao Tong University School of Medicine, Shanghai, China; ^3^Department of Immunology and Microbiology, Shanghai Jiao Tong University School of Medicine, Shanghai, China

**Keywords:** *Staphylococcus aureus*, dermal abscess, inhibition, nude mouse, phage JD007

## Abstract

**Aim:** In this study, *Staphylococcus* phage JD007 bactericidal activity and induced immune responses during treatment were assessed in a dermal abscess model.

**Materials and Methods:** Dermal abscesses in nude mice were established by injecting a clinical isolate of *S. aureus* SA325 isolated from the back under-dermal abscess of an in-patient.

**Results:** Phage JD007 was able to inhibit the growth of *S. aureus* SA325 at MOI = 1 or 10, significantly preventing the formation of dermal abscesses. Moderate immune responses were observed in the prevention group through detection of cytokines.

**Conclusion:** Phage JD007 inhibits the formation of dermal abscesses caused by a clinical *S. aureus* strain in nude mice without robust immune responses.

## Introduction

With the increasing prevalence antibiotic resistant infections, including MRSA (methicillin-resistant *Staphylococcus aureus*) and the emergence of VRSA (vancomycin-resistant *S. aureus*) (Xiao et al., 2013; Limbago et al., 2014), innovative treatments are urgently needed. Recently, there has been renewed interest in phage therapy (Thiel, 2004; Salmond and Fineran, 2015), which has been proposed as a potential antimicrobial therapy as far back as the 1920’s (Salmond and Fineran, 2015). Advances in biotechnology have led to the identification of numerous classes of bacteriophages with different host specificities such as K, GH15, Twort, phiIPLA-RODI, and phiIPLA-C1C (O’Flaherty et al., 2004; Kwan et al., 2005; Gu et al., 2012b; Gutierrez et al., 2015). Furthermore, whole genome sequences of these phages enables us to understand their biology at a much deeper level (Cui et al., 2017b).

Phages have been used to treat infectious disease in various animal models. A single dose of phage MR10 exhibited efficacy similar to linezolid in resolving the course of hindpaw infection in diabetic animals, suggesting this approach could serve as an effective strategy in treating MRSA mediated foot infections in diabetic individuals (Chhibber et al., 2013). Phage LS2a has been shown to prevent abscess formation in rabbits when injected simultaneously with *S. aureus* into the subcutaneous site (Wills et al., 2005). Phage Kpn5 could potentially be used as stand-alone therapy for the *Klebsiella pneumoniae* induced burn wound infection, effective even against antibiotic-resistant strains (Kumari et al., 2009). Additionally, in clinical trials, phage products have showed efficacy according to some reports (Marza et al., 2006).

Phage host specificity narrows their use with lysing efficacies that can vary significantly depending on the phage strain. Reports have also documented failures of phage therapy *in vivo* (Sarker and Brussow, 2016), so, it is necessary to evaluate phages’ bacteria killing efficacy both *in vitro* and in an animal model. *Staphylococcus* phage JD007, which belongs to family of *Myoviridae* was identified in 2012 and has been shown to have wide host range (Cui et al., 2012), capable of killing *S. aureus* of different MLST Types (Cui et al., 2017a). The aim of this study is to evaluate the treatment efficacy of JD007 in dermal abscesses caused by a clinical strain of MRSA and the immune responses elicited during therapy.

## Materials and Methods

### Animals

BALB/c nude mice (WT, Shanghai Super B&K laboratory animal Corp. Ltd., [SCXK(HU)2005—0001], Shanghai, China) were used in this study. Dermal abscesses were stereo tactically induced as described previously (Capparelli et al., 2007). Uninfected mice served as controls. Mice were euthanized on days 1, 3 and 10 after infection. Ethics eligibility of this study was approved by the Shanghai General Hospital Ethics Committee (Shanghai Jiao Tong University School of Medicine). All experiments were performed in accordance with the national animal protection guidelines approved by the local animal ethics committee. Fifty mice were randomly divided into five groups, 10 mice in each group, including control group (no treatment), phage JD007 group (injected with purified phage JD007), infection group (infected by *S. aureus* SA325), prevention group (phage JD007 were injected 1 h before mice was infected by *S. aureus* SA325), and treatment group (phage JD007 was injected 1 h after mice were infected by *S. aureus* SA325).

### Bacteriophage Purification

High-titre phage stocks were obtained through amplification in liquid LB (Luria-Bertani) medium supplemented with 10 μM MgCl_2_ and 5 μM CaCl_2_. In culture, *S. aureus* SA325 was infected phage JD007 at a MOI of 0.1 and incubated at 37°C overnight. The visible bacterial lysate in liquid culture were obtained, and then incubated with chloroform (final concentration was 2%) for 30 min with gentle shaking to kill residual bacteria. Remaining bacterial products were removed by centrifugation at 6,500 rpm (Beckman, JA18.0, United States) for 15 min. Phage contained in the supernatant was enriched at 4°C overnight using polyethylene glycol (PEG) 8000 (final concentration 10% w/v), and precipitated at 8,500 rpm for 20 min (Beckman, JA18.0, United States). Afterwards, the pellet was dissolved in TM buffer [(Tris-Mg^2+^ Buffer) 10 mM Tris–HCl (pH 7.2–7.5), 100 mM NaCl, 10 mM MgCl_2_.5 mM CaCl_2_] and vortexed. PEG 8000 was removed by adding the same volume of chloroform after vortexing. The solution was centrifuged at 4,000 *g*/min for 10 min, and the supernatant containing the phage was isolated. CsCl was added at a concentration of 0.5 g per 1 mL, and the phages were purified by discontinuous centrifugation in a CsCl gradient (1.33, 1.45, 1.50, and 1.70g/cm^3^) in TM buffer in Ultra-Clear tubes (Beckman Coulter, Inc., Fullerton, CA, United States) by centrifuging at 35000 rpm/min for 4 h. Finally, the layer with enriched phage was obtained with a syringe, dialyzed against TM buffer and stored at 4°C (Boulanger, 2009).

### *S. aureus* Strain and Culture Condition

*Staphylococcus aureus* strain SA325 (MRSA, confirmed by antibiotic resistant profile) was isolated from an in-patient with a re-occurring back under-dermal abscess due to long-term bed rest. SA325 concentration was calculated by serial dilutions when grown to OD_600nm_ = 0.4, and plated uniformly using the glass spreading rod, and cultured overnight at 37°C, the value of SA325 per OD_600nm_ was 9.5 × 10^9^ CPU/mL.

### *S. aureus* Growth Inhibition Assay

Drop tests were conducted using two-layer agar plates. *S. aureus* SA325 was cultured to OD_600nm_ = 0.4 in TSB (Tryptic soy broth) supplemented with 10 μM MgCl_2_ and 5 μM CaCl_2_. Bacteria grown to logarithmic phase were mixed with 0.7% agar LB and then poured on the plate of LB agar (1.5%) uniformly, incubating at room temperature for 30 min. Finally, 3 μL of serial dilutions of phage JD007 was dropped on the plate. The inhibition zones were observed after an overnight culture.

The inhibition growth curves for phage JD007 infected *S. aureus* SA325 were determined using MOIs of 0, 0.01, 0.1, and 1 inoculated in 96 well plates separately and incubated at 37°C. OD_600nm_ of these cultures was measured every 30 min extending for 8 h. Assay was performed three times (*n* = 3) with three biological replicates.

### Mice Dermal Abscess Infection Model

*Staphylococcus aureus* was prepared by culturing at 37°C in TSB to exponential phase at which time the bacteria was collected and washed twice with PBS with immediate centrifugation. SA325 was re-suspended at final concentration of 1 × 10^9^ CFUs/100μL, and kept on ice before injecting nude mice intradermally.

All animal experiments were approved by the Shanghai General Hospital Ethics Committee (Shanghai Jiao Tong University, China), and conducted according to the Chinese Law for Animal Protection. To investigate the antimicrobial efficacy of phage JD007 for subcutaneous abscesses, BALB/c nude mice (6–8 weeks old) were anesthetized by intraperitoneal injection of a saline solution containing fentanyl (0.05 mg/kg), midazolam (5 mg/kg), and medetomidine (0.5 mg/kg). The backs of the animals were disinfected with 70% ethanol, and 50 μL suspension containing 5 × 10^8^ CFU *S. aureus* SA325 in PBS were inoculated subcutaneously; 50 μL of phage JD007 was injected into the infected region. Mice were weighed before inoculation. Weight and abscess formation were measured daily extending for 10 days. The length (L) and width (W) of abscesses were determined using a caliper. The size of the abscesses was then calculated with the standard formula for area: V = [π/2] × L × W (Weinandy et al., 2014).

### Immune Response During Treatment Using *Staphylococcus* Phage JD007

Three mice in each described above were randomly enrolled and euthanized on the third day of the experimental protocol. Serum from each mouse was obtained, and immune cytokines (IL-1β, IL-6, IL-8, IFN-γ, and TNF-α) were measured using ELISA (Beijing 4A Biotech Co., Ltd.).

### Statistical Analysis

A two-tailed unpaired Student’s *t-*test was used for statistical analysis with GraphPad Prism Software. *P-*values of less than 0.05 were considered significant unless specifically indicated otherwise.

## Results

### *S. aureus* SA325 Growth Is Inhibited by Phage JD007 *in Vitro*

**FIGURE 1:**
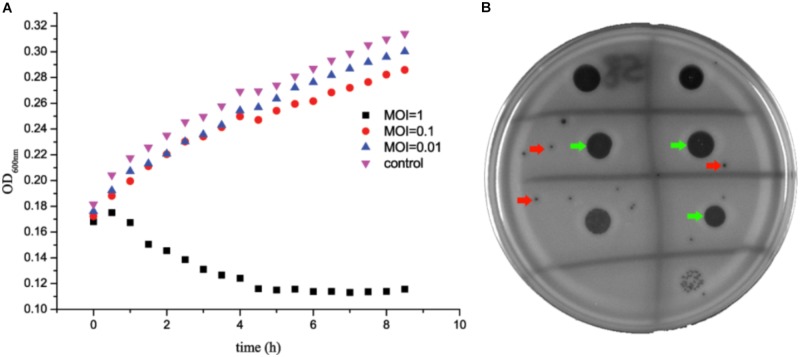
*Staphylococcus aureus* SA325 growth was inhibited by phage JD007 *in vitro*. **(A)** Phage JD007 could inhibit the growth of SA325 at MOI = 1. **(B)** Inhibition zones and plaques were formed by phage JD007 in two-layer agar plate of SA325 and were marked using green and red arrows, respectively.

*Staphylococcus aureus* SA325 was co-cultured with phage JD007 at 37°C with co-culture OD_600nm_ measurements made at 30 min intervals for 8 h. Results showed that phage JD007 inhibited the growth of *S. aureus* SA325 as early as 1 h after co-incubation at MOI = 1 (**Figure 1**). Whereas, *S. aureus* SA325 grew normally when infected by phage JD007 at lowers MOIs = 0.1 and 0.01. The drop test results showed that phage JD007 formed clear inhibition zones in two-layer agar plates.

### Phage JD007 Prevents *S. aureus* SA325-Mediated Dermal Abscess Formation in Nude BALB/c Mice *in Vivo*

**FIGURE 2:**
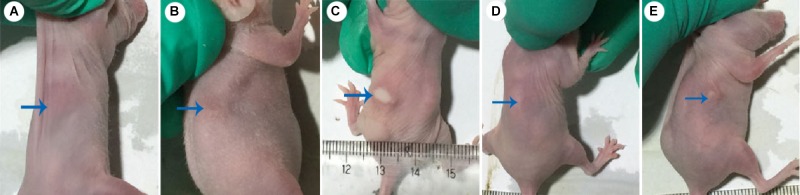
Photos of dermal abscesses in nude BALB/c mice using phage JD007. **(A–E)** Represented the dermal abscesses of mice individually in control group, phage group, infection group, prevention group, and treatment group. The blue arrows showed the skin sites injected with *S. aureus* SA325.

**FIGURE 3:**
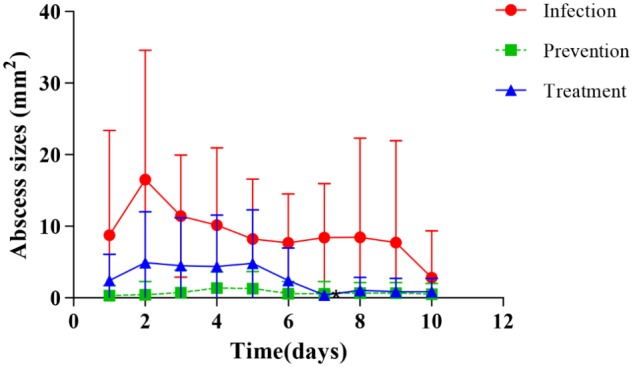
Changes of dermal abscesses sizes formed in nude BALB/c mice during phage therapy. There are no abscesses formed in control and phage groups during the whole procession of observation periods.

Dermal abscess formation was established in the back of nude mice and observed daily for 10 days. Results showed that the maximum sizes of dermal abscesses were achieved on the second day following infection. On the seventh day, all of the abscesses were scarred. As shown in **Figures 2, 3**, we could see no visible dermal abscesses formed in control group, phage group, and prevention group during the entire observation period. On the third day, the abscess sizes tended to decrease in size, and mice’s dermal abscess size in the treatment group were significantly smaller than those in the infection group (*p* < 0.05).

### Inflammatory Cytokines Responses of Nude BALB/c Mice During Phage Therapy

**FIGURE 4:**
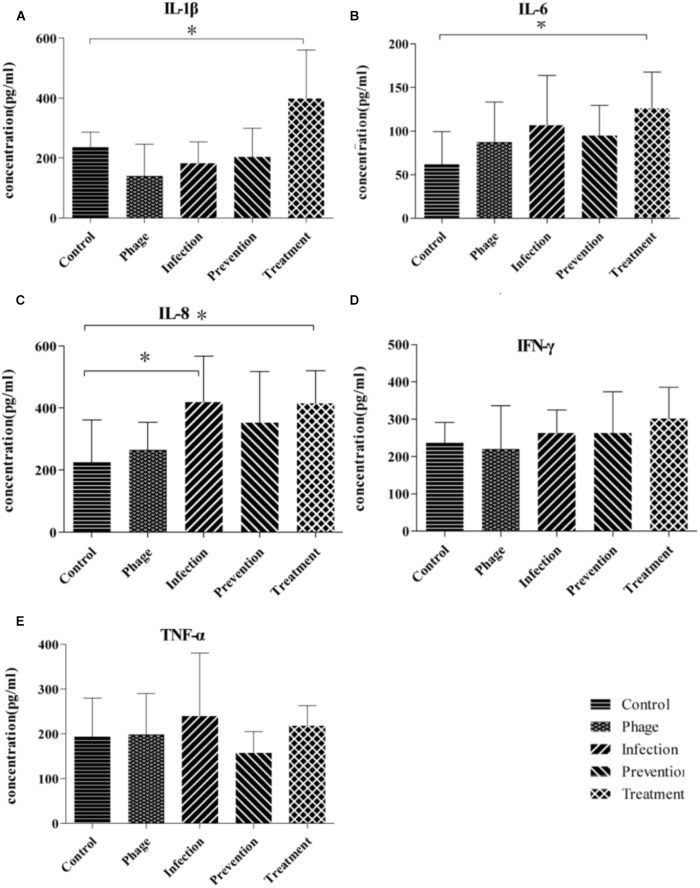
Inflammatory cytokines responses in nude BALB/c mice during phage therapy. **(A–E)** Are separately represents the sera level of IL-1β, IL-6, IL-8, IFN-γ, and TNF-α of mice from different groups, ^∗^*p* < 0.05.

Cytokines of IL-1β, IL-6, IL-8, IFN-γ, and TNF-α in the sera were measured using ELISA on the third day. As shown in **Figure 4**, there were no significant differences between IFN-γ and TNF-α among those groups. IL-1β, IL-6, and IL-8 in treatment groups were higher than those in control group, whereas there were no significant differences between the negative control and prevention groups.

### Weight of Nude BALB/c Mice During Phage Therapy

**FIGURE 5:**
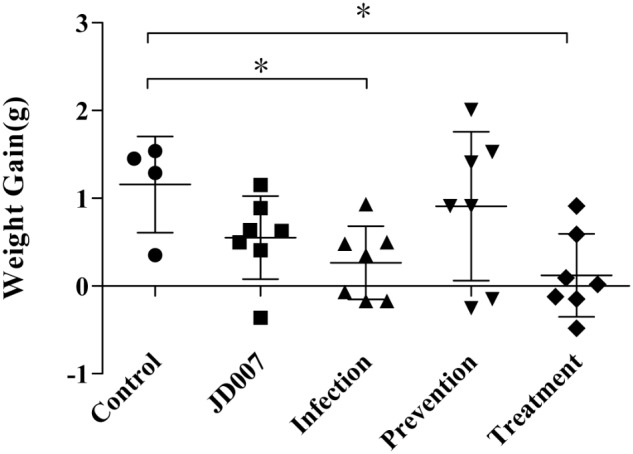
Weights gains in nude BALB/c mice during phage therapy. Body weights gain were calculated by subtracting original body weight from weights on the 10th day. Body weights gains in groups of infection and treatment were significantly lower than that in control group, ^∗^*p* < 0.05.

Weights of mice in each group were measured daily, encompassing the entire observation period. Body weights gain were calculated by subtracting original body weight from weights on the tenth day. As shown in **Figure 5**, weight gains in infection group and phage treatment were much lower than that compared with control group. The weight gain in the prevention and phage JD007 groups had no significant difference compared with the PBS control group during treatment.

## Discussion

*Staphylococcus aureus* SA325 was isolated from a re-occurring back under-dermal abscess of an in-patient with long-term bed rest. *In vitro* bactericidal activity tests showed that phage JD007 could form a clear inhibition zones using a two-layer soft agar plate assay, and could inhibit growth of SA325 at MOI = 1 or 10. In order to evaluate the ability of phage JD007 to prevent or treat of abscesses caused by *S. aureus*, a dermal abscess model was established in nude mice model using clinical isolate SA325, as reported previously (Malachowa et al., 2013). In this study, we successfully established the dermal abscess infection in nude BALB/c mice using *S. aureus* SA325. Furthermore, the efficacies of treatment of the abscesses using phage JD007 were evaluated. There were no observed abscess formation in the prevention group. The average abscess sizes of phage treatment group were smaller than those of the infection group. The results indicate that phage JD007 prevents formation of abscess. Also, it supports earlier observations on phage M^Sa^ inhibition *S. aureus* dermal abscess formation (Capparelli et al., 2007).

To evaluate the response of mice during prevention and treatment after phage JD007 inoculation, the weight changes were observed during the whole process. As bacteriophages are viruses containing nucleic acid and proteins, they may bring some risk to humans when used for the treatment of bacteremia, as the complex composition of the phage may induce unpredictable immune responses when administered to the blood. The average weights gains in the infection and treatment groups were significantly lower than those in the control, PBS, phage JD007, and prevention groups. Furthermore, immune responses of the mice during treatment were evaluated, mice sera was taken on the third day, and the inflammatory cytokines of IL-1β, IL-6, IL-8, IFN-γ, and TNF-α in serum were measured using ELISA. The cytokines IL-1β and IL-6 both belong to endogenous pyrogens, and they can be applied to the hypothalamus regulating center to cause fever. IL-1β and IL-6 levels were significantly higher in the treatment group, while there were no significant differences among prevention, infection, or control groups, indicating that phage JD007 treatment may destroy the cells of *S. aureus* robustly, leading to increase of IL-1β and IL-6 that may cause fever. It’s reported that TRIM29 could negatively regulate the proinflammatory cytokine IL-6 and TNF-α production in response to LPS and bacteria *Haemophilus influenzae* (Xing et al., 2016). Whether *S. aureus* infection suppresses the TRIM 29 expression and leads to increased expression of IL-6 should be further examined. IL-8 was significantly elevated in infection and treatment groups. IL-8 can signal chemotactic neutrophil leukocytes and activate T cells in the infection sites, clearing the pathogen. IFN-γ and TNF-α can activate mononuclear macrophages, which can enhance the phagocytosis and killing functions; TNF-α can directly kill the cell infected by virus. There were no significant changes in any of these groups.

**FIGURE 6:**
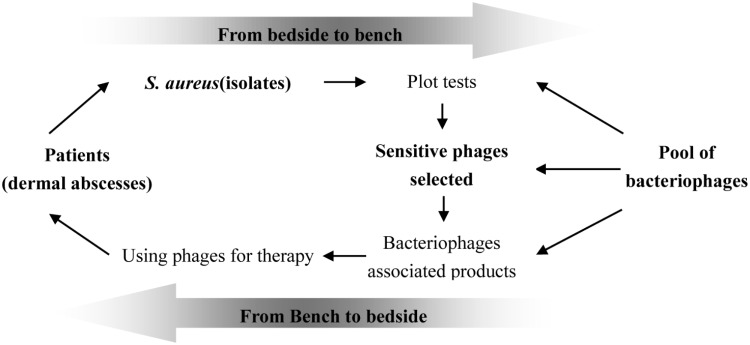
Proposed scheme of selecting phages for therapy. The success of phage therapy needs the pool of phages and their susceptibility to the definite infectious bacterial pathogen at infection sites should be tested previously; the products containing of different phages were produced firstly and the sensitive phages would be chosen for therapy.

The specificity of bacteriophages is one of important reason hindering clinical use of phage for therapy, though cocktails may overcome this fault (Gu et al., 2012a; Mendes et al., 2014). There are numerous public reports describing the success of phage therapy inhibiting infectious diseases caused by dermal associated infections (Vieira et al., 2012; Trigo et al., 2013). We postulate that phage therapy could become an important choice for the treatment of wound infections. As shown in **Figure 6**, the pool of different phages should be established before using them for therapy; at the same time, the bacteria causing infection should be isolated and its sensitivity to the phages in the pool confirmed. The formula of sensitive phages must be prepared at first; once the bacteria causing the infection is confirmed to be sensitive to phages in the pool, the formula containing the sensitive phage should be chosen for treatment of the infections. The success of phage therapy depends upon the pool of different kinds of bacteriophages.

In summary, phage JD007 could inhibit the growth of *S. aureus* both *in vitro* and *in vivo*. Phage JD007 can prevent abscess formation caused by *S. aureus* isolated from an in-patient’s abscess samples through under-dermal injection, and can also significantly reduce the severity of dermal abscess caused by *S. aureus*. In conclusion, phage JD007 is a promising candidate phage for use in preventing dermal abscess.

## Author Contributions

ZC, QTL, and BD carried out the experiments and drafted the manuscript. QTL, KD, BD, QZL, YZ, and MG participated in the mice model experiments. ZC, QTL, XG, and LL participated in the design of the study and performed the statistical analysis. ZC, QTL, and LL conceived the study, participated in its design, and coordinated to help draft the manuscript.

## Conflict of Interest Statement

The authors declare that the research was conducted in the absence of any commercial or financial relationships that could be construed as a potential conflict of interest.

## References

[B1] Boulanger P. (2009). Purification of bacteriophages and SDS-PAGE analysis of phage structural proteins from ghost particles. *Methods Mol. Biol.* 502 227–238. 10.1007/978-1-60327-565-1_13 19082559

[B2] Capparelli R. Parlato M. Borriello G. Salvatore P. Iannelli D. (2007). Experimental phage therapy against *Staphylococcus aureus* in mice. *Antimicrob. Agents Chemother.* 51 2765–2773. 10.1128/AAC.01513-06 17517843 PMC1932491

[B3] Chhibber S. Kaur T. Sandeep K. (2013). Co-therapy using lytic bacteriophage and linezolid: effective treatment in eliminating methicillin resistant *Staphylococcus aureus* (MRSA) from diabetic foot infections. *PLoS One* 8:e56022. 10.1371/journal.pone.0056022 23418497 PMC3572146

[B4] Cui Z. Feng T. Gu F. Li Q. Dong K. Zhang Y. (2017a). Characterization and complete genome of the virulent *Myoviridae* phage JD007 active against a variety of *Staphylococcus aureus* isolates from different hospitals in Shanghai, China. *Virol. J.* 14:26. 10.1186/s12985-017-0701-0 28179010 PMC5299689

[B5] Cui Z. Guo X. Dong K. Zhang Y. Li Q. Zhu Y. (2017b). Safety assessment of *Staphylococcus* phages of the family *Myoviridae* based on complete genome sequences. *Sci. Rep.* 7:41259. 10.1038/srep41259 28117392 PMC5259776

[B6] Cui Z. Song Z. Wang Y. Zeng L. Shen W. Wang Z. (2012). Complete genome sequence of wide-host-range *Staphylococcus aureus* phage JD007. *J. Virol.* 86 13880–13881. 10.1128/JVI.02728-12 23166274 PMC3503062

[B7] Gu J. Liu X. Li Y. Han W. Lei L. Yang Y. (2012a). A method for generation phage cocktail with great therapeutic potential. *PLoS One* 7:e31698. 10.1371/journal.pone.0031698 22396736 PMC3291564

[B8] Gu J. Liu X. Lu R. Li Y. Song J. Lei L. (2012b). Complete genome sequence of *Staphylococcus aureus* bacteriophage GH15. *J. Virol.* 86 8914–8915. 10.1128/JVI.01313-12 22843868 PMC3421715

[B9] Gutierrez D. Vandenheuvel D. Martinez B. Rodriguez A. Lavigne R. Garcia P. (2015). Two phages, phiIPLA-RODI and phiIPLA-C1C, lyse mono- and dual-staphylococcal biofilms. *Appl. Environ. Microbiol.* 81 3336–3348. 10.1128/AEM.03560-14 25746992 PMC4407228

[B10] Kumari S. Harjai K. Chhibber S. (2009). Efficacy of bacteriophage treatment in murine burn wound infection induced by *Klebsiella pneumoniae*. *J. Microbiol. Biotechnol.* 19 622–628. 19597322 10.4014/jmb.0808.493

[B11] Kwan T. Liu J. DuBow M. Gros P. Pelletier J. (2005). The complete genomes and proteomes of 27 *Staphylococcus aureus* bacteriophages. *Proc. Natl. Acad. Sci. U.S.A.* 102 5174–5179. 10.1073/pnas.0501140102 15788529 PMC556006

[B12] Limbago B. M. Kallen A. J. Zhu W. Eggers P. McDougal L. K. Albrecht V. S. (2014). Report of the 13th vancomycin-resistant *Staphylococcus aureus* isolate from the United States. *J. Clin. Microbiol.* 52 998–1002. 10.1128/JCM.02187-13 24371243 PMC3957794

[B13] Malachowa N. Kobayashi S. D. Braughton K. R. DeLeo F. R. (2013). Mouse model of *Staphylococcus aureus* skin infection. *Methods Mol. Biol.* 1031 109–116. 10.1007/978-1-62703-481-4_14 23824894

[B14] Marza J. A. Soothill J. S. Boydell P. Collyns T. A. (2006). Multiplication of therapeutically administered bacteriophages in *Pseudomonas aeruginosa* infected patients. *Burns* 32 644–646. 10.1016/j.burns.2006.02.012 16781080

[B15] Mendes J. J. Leandro C. Mottola C. Barbosa R. Silva F. A. Oliveira M. (2014). In vitro design of a novel lytic bacteriophage cocktail with therapeutic potential against organisms causing diabetic foot infections. *J. Med. Microbiol.* 63 1055–1065. 10.1099/jmm.0.071753-0 24869663

[B16] O’Flaherty S. Coffey A. Edwards R. Meaney W. Fitzgerald G. F. Ross R. P. (2004). Genome of staphylococcal phage K: a new lineage of *Myoviridae* infecting gram-positive bacteria with a low G+C content. *J. Bacteriol.* 186 2862–2871. 10.1128/JB.186.9.2862-2871.2004 15090528 PMC387793

[B17] Salmond G. P. Fineran P. C. (2015). A century of the phage: past, present and future. *Nat. Rev. Microbiol.* 13 777–786. 10.1038/nrmicro3564 26548913

[B18] Sarker S. A. Brussow H. (2016). From bench to bed and back again: phage therapy of childhood *Escherichia coli* diarrhea. *Ann. N. Y. Acad. Sci.* 1372 42–52. 10.1111/nyas.13087 27197768

[B19] Thiel K. (2004). Old dogma, new tricks–21st Century phage therapy. *Nat. Biotechnol.* 22 31–36. 10.1038/nbt0104-31 14704699

[B20] Trigo G. Martins T. G. Fraga A. G. Longatto-Filho A. Castro A. G. Azeredo J. (2013). Phage therapy is effective against infection by *Mycobacterium ulcerans* in a murine footpad model. *PLoS Negl. Trop. Dis.* 7:e2183. 10.1371/journal.pntd.0002183 23638204 PMC3636042

[B21] Vieira A. Silva Y. J. Cunha A. Gomes N. C. Ackermann H. W. Almeida A. (2012). Phage therapy to control multidrug-resistant *Pseudomonas aeruginosa* skin infections: in vitro and ex vivo experiments. *Eur. J. Clin. Microbiol. Infect. Dis.* 31 3241–3249. 10.1007/s10096-012-1691-x 22777594

[B22] Weinandy F. Lorenz-Baath K. Korotkov V. S. Bottcher T. Sethi S. Chakraborty T. (2014). A beta-lactone-based antivirulence drug ameliorates *Staphylococcus aureus* skin infections in mice. *ChemMedChem* 9 710–713. 10.1002/cmdc.201300325 24678014

[B23] Wills Q. F. Kerrigan C. Soothill J. S. (2005). Experimental bacteriophage protection against *Staphylococcus aureus* abscesses in a rabbit model. *Antimicrob. Agents Chemother.* 49 1220–1221. 10.1128/AAC.49.3.1220-1221.2005 15728933 PMC549253

[B24] Xiao M. Wang H. Zhao Y. Mao L. L. Brown M. Yu Y. S. (2013). National surveillance of methicillin-resistant *Staphylococcus aureus* in China highlights a still-evolving epidemiology with 15 novel emerging multilocus sequence types. *J. Clin. Microbiol.* 51 3638–3644. 10.1128/JCM.01375-13 23985906 PMC3889745

[B25] Xing J. Weng L. Yuan B. Wang Z. Jia L. Jin R. (2016). Identification of a role for TRIM29 in the control of innate immunity in the respiratory tract. *Nat. Immunol.* 17 1373–1380. 10.1038/ni.3580 27695001 PMC5558830

